# Drowning in Water, Not Symptoms: Extreme Hyponatremia Without Neurological Deficits

**DOI:** 10.51894/001c.143751

**Published:** 2025-09-30

**Authors:** Alex Pasculle, Sofia Tiberio, Harsh Y. Patel, Ryian Choudhury

**Affiliations:** 1 College of Osteopathic Medicine Michigan State University; 2 College of Osteopathic Medicine Michigan State University; 3 College of Osteopathic Medicine Michigan State University; 4 Medical Intensive Care Henry Ford Macomb

## 109

### BACKGROUND

Severe hyponatremia is a critical condition associated with significant morbidity and mortality, often presenting with altered mental status and neurological deficits. Hyponatremia below 100 mEq/L is exceedingly rare, with few reported cases. Beer potomania is a well-documented cause of profound hypotonic hyponatremia due to excessive alcohol consumption and inadequate solute intake. Chronic alcohol intake with poor nutrition leads to impaired renal water excretion due to low dietary solute load, resulting in severe dilutional hyponatremia. Here, we report the lowest documented serum sodium level to our knowledge (98 mEq/L), representing a rare case of profound hyponatremia without significant neurological symptoms or seizures.

### CASE PRESENTATION

A 39-year-old female with an unknown history presented with bilateral lower extremity weakness, muscle spasms, and shortness of breath. She was alert and oriented despite a sodium level of 98 mEq/L. Neurologic and musculoskeletal exams were unremarkable. Initial notable labs included potassium 2.9 mEq/L, chloride 60 mEq/L, creatinine 3.27 mg/dL (baseline 1.4), calcium 5.9 mg/dL, serum osmolality 224 mOsm/kg, and a BAC of 0.03 g%. Urine osmolality was 249 mOsm/kg, suggesting impaired free water excretion. Excessive alcohol intake with limited dietary solute intake suggested beer potomania. Her course was complicated by AKI, transaminitis, refeeding syndrome, and suspected aspiration pneumonia. Her sodium gradually corrected with the goal of 4-6 mEq/L per day with hypertonic saline, her neurological status remained intact, and she was eventually stabilized and transferred out of the ICU.

### DISCUSSION

This case demonstrates extreme hyponatremia with minimal neurological impairment, contrasting with previous reports of severe symptoms at similar sodium levels. Chronic alcohol use and malnutrition likely led to reduced renal solute excretion, predisposing her to profound hypotonic hyponatremia. The absence of significant neurological deficits despite such a low sodium level raises questions about adaptations in chronic hyponatremia and reinforces the importance of cautious sodium correction to avoid osmotic demyelination syndrome.

### CONCLUSION

This case highlights severe hyponatremia in beer potomania and alcohol-induced malnutrition and presents one of the lowest recorded sodium levels in a patient who remained largely asymptomatic.

